# Knowledge, Attitude and Practice on Pesticide Use among Vegetable Farmers in Madhyapur Thimi Municipality, Nepal: An Observational Study

**DOI:** 10.31729/jnma.9110

**Published:** 2025-06-30

**Authors:** Ranjan Suwal, Ajay Kumar Rajbhandari, Vijaya Laxmi Shrestha, Mahima Bataju, Mahesh Sharma, Bijay Khatri

**Affiliations:** 1 Department of Biochemistry, Patan Academy of Health Sciences, Lagankhel, Lalitpur, Nepal; 2 School of Public Health, Patan Academy of Health Sciences, Lagankhel, Lalitpur, Nepal; 3 Department of Public Health, National Academy for Medical Sciences, Old Baneshwor, Kathmandu, Nepal; 4 Department of Biochemistry, KIST Medical and Teaching Hospital, Gwarko, Lalitpur, Nepal; 5 Public Health Section, Health Service Division, Ministry of Social Development, Birendranagar, Surkhet, Karnali Province, Nepal; 6 Department of Public Health, Aarhus University, Aarhus C, Denmark

**Keywords:** *attitude*, *farmers*, *knowledge*, *pesticides use*, *practice*

## Abstract

**Introduction:**

Pesticides are groups of toxic chemical compounds designed to use for increased productivity by killing pest. Inappropriate handling of pesticides cause risk to targeted as well as non-targeted organisms and also humans. Consumption of the pesticides is increasing globally and in Nepal. The study aimed to study the existing knowledge, attitude and practices of pesticides use among vegetable farmers.

**Methods:**

A cross-sectional study was conducted among vegetable farmers who had used pesticides in the past 12 months for their farming. A household survey was conducted among 395 farmers from January to April 2023 in Madhyapur Thimi Municipality. Ethical approval was taken from the Ethical Review Board, Nepal Health Research Council (Reference number: 1696). A semi-structured questionnaire was developed from literature review and expert advices. The data was collected through face-to-face interviews. Data was analysed using descriptive statistics using SPSS 26.

**Results:**

The mean age of the farm workers was 50.73±11.1 years. Of all farmers 117 (29.62%) were illiterate and 355 (89.87%) farmers received information of pesticides from friends and family. A total of 348 (88.1%) farmers had never received training on pesticide use. However, only 132 (33.41%) farmers always used to wear proper personal protective equipment in practice. Headache was the most common 57 (69.51%) health adverse symptom after pesticides application.

**Conclusions:**

Our study shows that farmers have limited knowledge about pesticides and use them in unsafe ways.

## INTRODUCTION

Pesticides by definition are toxic chemical agents predominantly used in agriculture to increase productivity by preventing or controlling pests, disease, weeds, and other plant pathogens.^1^ If inappropriately handled, they usually are capable of harming all forms of life other than the targeted pest species.^2^

Globally, 50% of the world labour is employed in agriculture.^3^ Pesticide use has resulted in thousands of cases of acute and chronic poisoning world-wide, with effects of varying severity on human health, from mild effects to death.^4^ In Nepal, there has been a more than six fold increase in the import and consumption of chemical pesticides over years from 1997/98 to 2011/2012.^5^

In Nepal, vegetable farmers are more susceptible to pesticide exposure, as 90% of the total pesticides are used is in vegetable farming.^6^ Hence, this study aimed to identify the existing knowledge, attitude and practices of pesticides use among vegetable farmers.

## METHODS

A cross-sectional quantitative study was conducted among vegetable farmers in Madhyapur Thimi municipality of Bhaktapur, Nepal from January, 2023 to April, 2023. Farmers of Madhyapur Thimi Municipality was chosen as it is considered as one of the largest vegetables suppliers of the Kathmandu Valley. Out of 1,147-hectare of municipality area, 802-hectare land is cultivable and 60% of cultivable land has irrigation facility.

This household survey included farmers engaged in vegetable farming for the past 12 months who used commercially available pesticides. The study population consisted of the primary family member responsible for pesticide application in each household.

The sample size for this study was calculated using a single population formula appropriate for a crosssectional study design. With a 95% confidence level and a 5% margin of error, and 50% prevalence (p), the initial sample size was 385. Considering 5% nonresponse rate, the final sample size was 405. According to the municipal report, there were 709 households engaged in commercial vegetable farming across 9 wards. We used probability proportionate to size sampling to determine the sample size for each ward and selected clusters within each ward to survey the households.

The semi-structured questionnaire in Nepali language was developed from expert advices and literature review.^7-10^ Questionnaires were divided into A, B, C, D and E sections. The section A covered sociodemographic information, farming practices, and the types of pesticides used. The B, C and D sections were related to the respondents' knowledge, attitude, and practices, respectively, regarding use of pesticide. The section E consisted of self-reported health impacts of pesticides on respondents. The questionnaire in Nepali language was pre-tested among 20 vegetable farmers in Bhaktapur municipality.

The data was collected through face-to-face interviews using the questionnaire by four trained enumerators with a background in public health education and fluency in local Newari language. The enumerators received comprehensive one-day training to ensure uniform methods for approaching potential participants, explaining the nature of the information needed, obtaining consent, extracting information through interviews, and recording the information on the semi-structured questionnaire. The same enumerators were also involved in the pre-testing.

The completed questionnaires were checked daily for completeness, legibility, and consistency. Data entry was done using Microsoft Excel 2019 and analyses were done using IBM Statistical Package for Social Science (SPSS) 26.0 software. The frequencies and percentage were computed to assess the distribution of sample characteristics.

Ethical approval was taken from the Ethical Review Board of Nepal Health Research Council (Reference number: 1696). Participation was voluntary, receiving no incentives for participating in the study. Verbal consent was taken from illiterate participants and written informed consent was obtained from literate ones.

## RESULTS

There were 338 (85.56%) and 342 (86.58%) respondents were aware that information on pesticide labels should be read and appropriate pesticides should be selected for a specific pest. In contrast, 129 (32.65%), 162 (41.01%) and 214 (54.17%) didn't know about potential hazard identification, pesticide poisoning symptoms and registered pesticide products discrimination for use respectively (Table 1).

**Table 1:** Knowledge on pesticide use (n=395).

Statement	Yes n(%)	No n(%)	Don't Know n(%)
Information on pesticide labels should be read and well understood	338(85.56)	22(5.56)	35(8.86)
Appropriate pesticide products should be selected for a specific pest problem	342(86.58)	7(1.77)	46(11.64)
Appropriate weather conditions should be considered for pesticide application	382(96.70)	1(0.25)	12(3.03)
Correct application rate should be defined for a specific product	330(83.54)	8(2.02)	57(14.43)
Potential hazards should be identified for different pesticide formulations	258(65.31)	8(2.02)	129(32.65)
Symptoms of pesticide poisoning in the operator should be recognized	222(56.20)	11(2.78)	162(41.01)
Registered pesticide products should be discriminated as required for use	169(42.78)	12(3.03)	214(54.17)
Use of personal protective equipment is not necessary during pesticides application	31(7.84)	356(90.12)	8(2.02)
Disposal of empty pesticides container at proper place is not necessary	42(10.63)	337(85.31)	16(4.05)
Hands washing is necessary after pesticides application	371(93.92)	3(0.75)	21(5.31)

**Source of information about pesticide**

Among various sources of information about pesticides, 355 (89.87%) farmers received information from their friends and family. Similarly, 255 (64.55%) farmers reported receiving information from neighbours, 177 (44.81%) farmers from agrovets, 31 (7.84%) farmers from social media, 27 (6.83%) farmers from NGOs, 24 (6.07%) farmers from television, and 22 (5.56%) farmers from FM radio.

**Attitude towards pesticide use**

A total of 272 (68.85%) respondents didn't believe that crop yield would drop if pesticide is not overused. However, 37 (9.36%) were neutral and 86 (21.76%) agreed on the statement. Similarly, 353 (89.36%) agreed that protection is necessary to avoid harm due to pesticides. Furthermore, 226 (57.21%) of participants disagreed that reducing the amount of pesticides would not mitigate environmental pollution whereas 105 (26.58%) had no idea about it and 64 (16.19%) agreed on the statement. Additionally, 279 (70.62%) participants disagreed that spraying an additional amount of pesticides wouldn't reduce the price and quality of products. (Table 2)

**Practices on pesticides use**

All respondents reported using some form of pesticide in their vegetable farming. Insecticide was the most commonly used pesticide, reported by 384 (97.21%) respondents, followed by biopesticides, which were used by 64 (16.2%) respondents. Other pesticides included fungicides by three (0.75%) respondents, bactericides by one (0.25%) respondent, herbicides by eight (2.02%) respondents, and rodenticides by four (1.01%) respondents.

A total of 329 (83.29%) respondents applied pesticides for 4 to 6 months per year in vegetable farming, while 47 (11.89%) respondents applied pesticides for less than 3 months, and 19 (4.81%) respondents sprayed for 7-9 months.

A total of 348 (88.1%) respondents had never received training on pesticide use. Meanwhile, 15 (3.79%) farmers had received training more than five years ago, 23 (5.82%) farmers had received training in the past 1-5 years, and only 9 (2.27%) farmers had received training in the past year.

Only 24 (6.07%) respondents always read the safety precautions before handling pesticides, and 50 (12.65%) always followed the safety precautions and 30 (7.59%) never did. A total of 132 (33.41%) farmers always wore PPE (Personal Protective Equipments) during pesticide application, 140 (35.44%) wore it often and 21 (5.21%) never wore PPE. In addition, 239 (60.5%) respondents never use pesticides beyond the required amount. Interestingly, more than 80% of participants always avoided smoking and eating during application, always washed their hands immediately after spraying, always stored pesticides in sealed and locked containers, always store pesticide products in safe places, always discouraged vulnerable family members from handling spraying and always harvest vegetables at least after 4 days of last spraying. Similarly, 283 (71.64%) participants always preferred pesticides with less toxicity.

In contrast, participants were found to have poor practices such as always changing clothes immediately after spraying by only 139 (35.18%), always washing used clothes separately from others by only 96 (24.3%), always showered immediately after spraying by only 66 (16.7%) and always disposed container according to pesticide label by only 151 (38.22%). The very poor practice was found in always displaying the signboard or red flag in the spraying area for warning by 31 (7.84%) respondents while 348 (88.1%) never followed it. (Table 3)

**Table 2:** Attitude towards pesticide use (n=395).

Statement	5 (Strongly Agree) n(%)	4 (Agree) n(%)	3 (Neither Agree Nor Disagree) n(%)	2 (Disagree) n(%)	1 (Strongly Disagree) n(%)
If pesticide is not overused, crop yield would considerably drop	45(11.39)	41(10.37)	37(9.36)	230(58.22)	42(10.63)
Pesticides would substantially harm health, if necessary, protection is not followed while handling pesticide	245(62.02)	108(27.34)	23(5.82)	16(4.05)	3(0.75)
Environmental pollution would not be mitigated by decreasing the pesticide amount being sprayed	18(4.55)	46(11.64)	105(26.58)	197(49.87)	29(7.34)
Spraying additional pesticide amounts would not reduce the product quality or price	14(3.54)	18(4.55)	84(21.26)	224(56.7)	55(13.92)

**Table 3:** Practices on pesticides use (n=395).

Statement	5(Always) 4(Often)	3 (Sometimes) n(%)	2 (Rarely) n(%)	1 (Never) n(%)
I read the safety precautions of the pesticide label before handling	24(6.07)	85(21.51)	96(24.30)	113(28.6)	77(19.49)
I follow the safety precautions of the pesticide label during handling	50(12.65)	161(40.75)	100(25.31)	54(13.67)	30(7.59)
I wear proper personal protective equipment/clothing during application (masks, gloves, long-sleeved body cover and gum boots)	132(33.41)	140(35.44)	85(21.51)	17(4.30)	21(5.31)
I do not use pesticide products beyond the required amount	239(60.50)	97(24.55)	37(9.36)	17(4.30)	5(1.26)
I avoid smoking cigarettes or eating food during application	317(80.25)	25(6.32)	5(1.26)	9(2.27)	39(9.87)
I wash my hands immediately after spraying pesticides.	341(86.32)	39(9.87)	8(2.02)	5(1.26)	2(0.50)
I change clothes immediately after spraying pesticides	139(35.18)	36(9.11)	59(14.93)	79(20.00)	82(20.75)
I wash clothes used for spraying separately from ordinary clothes	96(24.30)	37(9.36)	70(17.72)	99(25.06)	93(23.54)
I shower immediately after spraying pesticides	66(16.70)	13(3.29)	80(20.25)	111(28.10)	125(31.64)
I dispose empty containers in accordance with the pesticide label	151(38.22)	92(23.29)	98(24.81)	31(7.84)	23(5.82)
I store pesticide products sealed and locked in original containers	319(80.75)	54(13.67)	11(2.78)	5(1.26)	6(1.51)
I store pesticide containers in the safe place (dry, away from light, not accessible to children)	330(83.54)	51(12.91)	5(1.26)	1(0.25)	8(2.02)
I prefer pesticides that have less toxicity	283(71.64)	65(16.45)	32(8.10)	8(2.02)	7(1.77)
I display a signboard or red flag in the sprayed area for warning	31(7.84)	1(0.25)	9(2.27)	6(1.51)	348(88.10)
I discourage vulnerable family members (pregnant, under five children, those with chronic diseases like COPD, CKD) to handle pesticides	335(84.81)	30(7.59)	3(0.75)	2(0.50)	25(6.32)
I harvest vegetables at least after four days of last spraying	347(87.84)	30(7.59)	12(3.03)	4(1.01)	2(0.50)

**Table 4:** Socio-demographic characteristics of the respondents (n=395).

Characteristics	n (%)
Age in years	
25-34	19 (4.81)
35-44	99 (25.06)
45-54	137 (34.68)
55-64	88 (22.27)
65-74	46 (11.64)
75-84	6 (1.51)
**Sex**	
Male	276 (69.87)
Female	119 (30.12)
**Educational status**	
Illiterate	117 (29.62)
Primary and lower secondary	244 (61.77)
Secondary and higher secondary	23 (5.82)
Bachelors and above	11 (2.78)
**Family type**	
Nuclear	260 (65.82)
Joint/Extended	135 (34.17)
**Monthly Income from Vegetable Farming (USD)**	
< 43	48 (12.15)
43-130	117 (29.62)
130-215	137 (34.68)
215-307	75 (18.98)
307-423	11 (2.78)
423-846	3 (0.75)
>846	4 (1.01)
**Size of farmland for vegetable farming in hectare**	
Less than 0.10	223 (56.45)
0.10 to 0.25	156 (39.49)
More than 0.25	16 (4.05)
**Types of farmland ownership**	
Own	202 (51.13)
On lease	89 (22.53)
Own and on lease	104 (26.32)
**Family involvement**	
Full time	292 (73.92)
Part time	103 (26.07)

**Symptoms of health issues after applying pesticides**

After the application of pesticides, 82 (20.75%) respondents reported experiencing symptoms related to health issues. Among those who developed symptoms, the most prevalent was headaches, affecting 57 (69.51%) respondents. Other reported symptoms included eye irritation in 43 (52.43%) respondents, dizziness in 14 (17.07%) respondents, skin irritation in 14 (17.07%) respondents, and vomiting in 5 (6.09%) respondents. Out of 395 respondents, the mean age was 50.73±11.0 years with mean age of male and female were 50.71±11.02 and 50.77±11.31 years respectively. A total of 276 (69.87%) participants were male, and 119 (30.12%) were female. A total of 117 (29.62%) respondents were illiterate, 135 (34.17%) lived with extended family, 302 (76.45%) respondents monthly income is less than 215 USD, and 223 (56.45%) respondents were using less than 0.10 hectare land. The number of farmers using their own land were 202 (51.13%) and 292 (73.92%) respondents were involved full time. (Table 4)

## DISCUSSION

All respondents reported using some form of pesticide in their vegetable farming, with insecticides being the most commonly used (97.2%). Less than nine out of ten (89.87%) respondents received information about pesticides from their friends and family, and a similar proportion (88.1%) of respondents had never received training on pesticide use.

All respondents reported using some form of pesticide in their vegetable farming in our study as well as in a study in Bara and Dhading.^11^ Higher dependency on chemical pesticides was reported in other parts of Nepal too.^12^ While chemical pesticides may contribute to short-term crop yields, their negative long-term effects on soil health, biodiversity, and human well-being have been widely documented.^4^ This ever growing dependency on pesticides raises significant concerns about the environmental and health implications, not only for farmers but also for consumers.

Our study revealed significant gaps in knowledge regarding the selection of appropriate pesticides and their potential hazards. Another study in Bhaktapur, Nepal, found that farmers' knowledge of various aspects of pesticide use—including handling, selection, potential harmful effects, and proper application was inadequate.^13^ In contrast, a study conducted in Panchkhal, Kavreplanchowk, another key hub of vegetable farming in Nepal, found that the majority of farmers (83%) had good knowledge about correct use of pesticide.^14^ The same study highlights that the municipality's annual awareness programs may have played a crucial role in fostering higher levels of pesticide knowledge among farmers.^14^

The Government of Nepal has established an agricultural extension system at both the district and local levels, designed to provide farmers with technical support and guidance on crop production, pest management, and protection practices.^15^ However, our study showed that majority relied on friends, family, neighbours and Agrovets for information regarding pesticide use, and none mentioned about the government or local bodies. A study in Chitwan, Nepal also has reported that more than 90% farmers relied on local pesticide retailers for technical know-how about pesticide selection, handling, and use.^16^ Agrovet employees, in general, lack formal technical training, which often leads to the dissemination of misleading or inaccurate information.^16^ Their focus on potentially prioritizing product sales could compromise the objectivity of the advice given to farmers. Therefore, it is crucial that agrovet employees receive proper training or orientation on pesticide safety, handling, and application. Alternatively, regulatory measures should be implemented to ensure that agrovet not only sell pesticides but also provide accurate, science-based information to farmers. Such regulations could include mandatory education programs or certifications for employees, alongside clear guidelines on responsible pesticide use and integrated pest management practices.

Training programs designed to increase awareness of the harmful effects of pesticides and improve safe handling practices have been shown to enhance the knowledge and practices of both agrovet retailers and farmers.^14,17^ However, our study found that 88% of farmers had never received training on pesticide use, which may have contributed to the poor knowledge and poor practices observed in our study group. This lack of training is not a new issue; a 2010 study similarly found that the majority of farmers (93%) had not received any formal training on pesticide use.^10^ One possible explanation for this persistent gap in knowledge could be the shifting landscape of Nepal's economy, where the agricultural sector has seen a decline in importance as migration for labour opportunities has increased. As the country moves away from its traditional agricultural base, the government's focus on improving agricultural practices has diminished, leaving farmers with limited access to the necessary resources, training, and support to implement safe and sustainable farming practices.

The pesticide storage was better than pesticides use and disposal in our study. Despite awareness of adverse health and environmental effects, proper safety measures and disposal methods are not consistently practiced in other studies too.^13,16^

In our study, one-fifth of the farmers reported experiencing one or more symptoms related to health issues following pesticide use. Among those affected, the most common symptoms were headaches, which were reported by a significant portion of the participants, followed by eye irritation and dizziness. These findings are consistent with a study conducted in Chitwan, Nepal, where 18.7% of farmers experienced acute health symptoms after handling chemical pesticides.^18^ Similar symptoms, including dizziness, headaches, skin allergies, and eye irritation, were observed in that study,^18^ highlighting a common pattern of adverse health effects linked to pesticide exposure across different regions. This parallel suggests that pesticide use poses a widespread health risk to farmers, regardless of geographical location. Moreover, our study revealed poor pesticide use practices, including improper handling and inadequate protective measures. These unsafe practices have been consistently linked to health problems in various studies conducted in Nepal, underscoring the significant risks associated with improper pesticide application.^18-20^

The study was limited to vegetable farmers in a municipality and cannot be generalized to farmers growing other crops or those in other parts of the country. Additionally, the study relied on self-reported data from farmers, which may be subject to recall bias or social desirability bias.

## CONCLUSIONS

Our study highlights inadequate knowledge of pesticides and unsafe application practices, which pose significant health risks to both farmers and the environment. Addressing these issues through targeted education and training programs is crucial to improving pesticide handling practices, enhancing farmers' knowledge, and ultimately reducing the adverse impacts of pesticide use on health and the ecosystem.

## References

[1] Pathak VM, Verma VK, Rawat BS, Kaur B, Babu N, Sharma A (2022). Current Status of Pesticide Effects on Environment, Human Health and it's Eco-Friendly Management as Bioremediation: A Comprehensive Review.. Frontiers in microbiology..

[2] Kaur R, Choudhary D, Bali S, Bandral SS, Singh V, Ahmad MA (2024). Pesticides: An Alarming Detrimental to Health and Environment.. Science of The Total Environment..

[3] International Labour Organization. Chemicals in Agriculture Geneva, Switzerland 2023..

[4] Ahmad MF, Ahmad FA, Alsayegh AA, Zeyaullah M, AlShahrani AM, Muzammil K (2024). Pesticides Impacts on Human Health and the Environment with their Mechanisms of Action and Possible Countermeasures.. Heliyon..

[5] Aryal KK NS, Lohani GR, Jors E, Neupane D, Khanal PR, Jha BK, Dhimal M (2016). Health Effects of Pesticide among Vegetable Farmers and the Adaptation Level of Integrated Pest Management Program in Nepal, 2014..

[6] Atreya K, Sitaula BK (2010). Mancozeb: Growing Risk for Agricultural Communities?. Himalayan Journal of Sciences..

[7] Mohanty MK, Behera BK, Jena SK, Srikanth S, Mogane C, Samal S (2013). Knowledge Attitude and Practice of Pesticide use Among Agricultural Workers in Puducherry, South India.. Journal of forensic and legal medicine..

[8] Atreya K, Sitaula BK, Overgaard H, Bajracharya RM, Sharma S (2012). Knowledge, attitude and practices of pesticide use and acetylcholinesterase depression among farm workers in Nepal.. International journal of environmental health research..

[9] Gesesew HA, Woldemichael K, Massa D, Mwanri L (2016). Farmers Knowledge, Attitudes, Practices and Health Problems Associated with Pesticide Use in Rural Irrigation Villages, Southwest Ethiopia.. PloS one..

[10] Shrestha P, Koirala P, Tamrakar AS (2010). Knowledge, Practice and Use of Pesticides among Commercial Vegetable Growers of Dhading District, Nepal.. Journal of Agriculture and Environment..

[11] Mainali RP, Thapa RB, Tiwari S, Pokhrel P, Ansari AR (2014). Knowledge and Practices on Eggplant Fruit and Shoot Borer, Leucinodes orbonalis Guenee Management in Dhading and Bara Districts of Nepal.. Albanian Journal of Agricultural Sciences..

[12] Jyoti S, Lama P, Yadav A, Vaidya S, Joshi SK (2023). Knowledge, Attitude, and Practice of Pesticide use among Agricultural Workers of Lamatar Village Development Committee, Lalitpur District: A cross-sectional study.. International Journal of Occupational Safety and Health..

[13] Thapa S, Piras G, Thapa S, Goswami A, Bhandari P, Dahal B (2021). Study on Farmers' Pest Management Strategy, Knowledge on Pesticide Safety and Practice of Pesticide use at Bhaktapur District, Nepal.. Cogent Food & Agriculture..

[14] Paudel L, Neupane R, Shrestha L, Budhathoki L, Paudel KR (2024). Knowledge on Chemical Pesticide use among Farmers Exposed to Pesticides in Panchkhal Municipality, Kavrepalanchok, Nepal.. Advances in Public Health..

[15] Tamang K, Neupane A, Jaishi M (2020). Agricultural Extension Service Delivery in Provincial and Local Government of Nepal: an Integrative Literature Review.. Journal of te Institute of Agriculture and Animal Science..

[16] Rijal JP, Regmi R, Ghimire R, Puri KD, Gyawaly S, Poudel S (2018). Farmers' Knowledge on Pesticide Safety and Pest Management Practices: A Case Study of Vegetable Growers in Chitwan, Nepal.. Agriculture..

[17] Vaidya A, Gyenwali D, Tiwari S, Pande BR, Jrs E. (2017). Changes in Perceptions and Practices of Farmers and Pesticide Retailers on Safer Pesticide Use and Alternatives: Impacts of a Community Intervention in Chitwan, Nepal.. Environ Health Insights..

[18] Kafle S, Vaidya A, Pradhan B, Jrs E, Onta S (2021). Factors Associated with Practice of Chemical Pesticide Use and Acute Poisoning Experienced by Farmers in Chitwan District, Nepal.. International Journal of Environmental Research and Public Health..

[19] Lamichhane R, Lama N, Subedi S, Singh SB, Sah RB, Yadav BK (2019). Use of Pesticides and Health Risk among Farmers in Sunsari District, Nepal.. J Nepal Health Res Counc..

[20] Bhandari S, Paneru S, Pandit S, Rijal S, Manandhar HK, Ghimire BP (2020). Assessment of Pesticide use in Major Vegetables from Farmers' Perception and Knowledge in Dhading District, Nepal.. Journal of Agriculture and Natural Resources..

